# Complete genome sequences of 22 *Staphylococcus* spp. bacteriophages isolated from a single swine farm environment in Quebec, Canada

**DOI:** 10.1128/mra.00886-26

**Published:** 2026-09-24

**Authors:** Mousumi Sarker Chhanda, Eya Ladhari, Laura Zerillo, Valérie E. Paquet, Rébecca E. St-Laurent, Pierre-Étienne Marcoux, Audrey Turmel, Martine Denicourt, Marie-Ève Lambert, Antony T. Vincent, Steve J. Charette

**Affiliations:** 1 Institut de biologie intégrative et des systèmes (IBIS), Université Laval 213566, Quebec City, Quebec, Canada; 2 Département de biochimie, de microbiologie et de bio-informatique, Université Laval 4440, Quebec City, Quebec, Canada; 3 Centre de recherche en infectiologie porcine et avicole (CRIPA), Fonds de recherche du Québec, Nature et technologies 242137, Saint-Hyacinthe, Quebec, Canada; 4 Services Vétérinaires Ambulatoires Triple-V Inc., Acton Vale, Quebec, Canada; 5 Département de sciences cliniques, Faculté de médecine vétérinaire, Université de Montréal 574575, Saint-Hyacinthe, Quebec, Canada; 6 Département des sciences animales, Faculté des sciences de l'agriculture et de l'alimentation, Université Laval 177455, Quebec City, Quebec, Canada; Queens College Department of Biology, Queens, New York, USA

**Keywords:** bacteriophage, *Staphylococcus hyicus*, Québec, farm, water, manure, Twort

## Abstract

Twenty-two bacteriophages infecting *Staphylococcus* spp. were isolated from water and manure collected on a Quebec swine farm affected by exudative epidermitis. Phylogenetic analysis of their genomes showed that 20 of them clustered into two groups, Twort- and ShyM-like. No CRISPR-Cas systems, antimicrobial resistance determinants, or virulence genes were identified.

## ANNOUNCEMENT

*Staphylococcus hyicus* causes exudative epidermitis (EE), a skin infection affecting piglets with negative impacts on swine health (1–5). Rising antimicrobial resistance in swine production has increased interest in bacteriophages as an alternative to antibiotics for the control of *S. hyicus* infections (6–10).

We report the genome sequences of 22 lytic bacteriophages. Phages were isolated from drinking trough water and manure collected from enclosures housing piglets showing clinical signs of EE in a commercial swine farm in Quebec, Canada.

Phage isolation and propagation were performed using *S. hyicus* strains as hosts (Table 1). Manure samples (5 mL) were suspended in 40 mL of 1× phage buffer (50 mM Tris-HCl pH 7.5, 100 mM NaCl, 8 mM MgSO₄) and incubated overnight, then centrifuged (3,200 × *g*, 10 min) three times, and filtered using a 50 mL syringe with 0.45 µm filters, while water samples (50 mL) were centrifuged and filtered once. Filtered lysates (5 mL) were enriched with 5 mL of 2× tryptic soy broth (Wisent, Inc., Saint-Jean-Baptiste, Quebec, Canada) and exponentially growing host bacteria (100 µL) and incubated at 37°C for 24 h with shaking (150 rpm). Phages were detected by spot assays (11), and positive lysates were purified through three rounds of plaque isolation. High-titer stocks were obtained by amplification and stored at 4°C for short-term use and in 15% glycerol at −70°C for long-term preservation.

**TABLE 1:** Genomic characteristics of 22 *Staphylococcus spp*. bacteriophages isolated from a swine farm in Quebec, Canada^*a*^

Phage name	Host strain^*b*^	Genome size (bp)	Coverage (×)	GC content (%)	Total CDS	Lysis gene	Raw reads	Closest phage in GenBank	Percent identity (%)^*c*^	SRA accession number	NCBI accession number
BBQL5	SC369	139,223	255.1	30.47	247	6	448,290	VB_SauM	99.31	SRR38864792	PZ622584
BBQL6	SC369	139,096	525.6	30.47	247	6	1,923,974	VB_SauM	99.46	SRR38864787	PZ622585
BBQL4	SC392	139,095	953.4	30.47	248	6	2,350,651	VB_SauM	99.29	SRR38864785	PZ622583
BEQE3	SC369	139,071	2,432.5	30.47	247	6	10,296,776	VB_SauM	99.42	SRR38864788	PZ622586
BFQL1	SC392	139,071	5,433.7	30.47	247	6	10,597,131	VB_SauM	99.38	SRR38864811	PZ622596
BBQE1	SC369	138,893	264	30.47	249	6	501,254	VB_SauM	99.31	SRR38864784	PZ622580
BBQL1	SC361	138,860	358.5	30.46	248	6	476,244	VB_SauM	99.29	SRR38864783	PZ622582
BBQE2	SC369	138,834	312.7	30.47	248	6	571,044	VB_SauM	99.29	SRR38864813	PZ622581
BEQL1	SC392	137,299	2,320.4	30.44	241	6	9,052,526	VB_SauM	99.30	SRR38864803	PZ622587
BFQL10	SC373	134,303	548	30.23	234	7	304,066	Twort	98.13	SRR38864804	PZ622595
BQE4	SC361	134,284	2,640.6	30.44	235	7	2,730,321	Twort	98.13	SRR38864791	PZ166799
BFQE12	SC359	133,156	1,062.9	30.22	235	7	537,599	Twort	98.13	SRR38864809	PZ622591
BEQL8	SC388	133,011	440.9	30.25	230	7	199,294	Twort	98.13	SRR38864808	PZ622589
BFQE2	SC392	132,882	920.6	30.26	230	7	466,373	Twort	98.13	SRR38864807	PZ622592
BEQL9	SC388	132,668	457.1	30.25	231	7	208,552	Twort	98.13	SRR38864805	PZ622590
BFQE9	SC359	132,597	920.6	30.25	232	7	485,620	Twort	98.13	SRR38864810	PZ622594
BFQE4	SC369	132,595	220.3	30.26	230	7	333,211	Twort	98.13	SRR38864806	PZ622593
BQE1	SC392	132,468	6,811.8	30.25	230	7	6,289,201	Twort	98.13	SRR38864793	PZ166798
BQE7	SC386	132,467	1,775.7	30.26	227	7	1,979,832	Twort	98.13	SRR38864790	PZ166800
BFQL3^*d*^	SC369	116,985	216.9	31.31	160	2	9,530,609	SPbeta-like 2	76.58	SRR38864814	PZ622597
BQL4	SC361	115,356	1,196.8	30.28	182	7	4,358,476	Twort	98.13	SRR38864789	PZ166801
BEQL7	SC373	83,716	882.5	31.72	161	3	259,346	PMBT8	99.16	SRR38864794	PZ622588

^*a*^ Phage names ending with “E” indicate isolates recovered from water samples, whereas those ending in “L” were isolated from manure samples.

^*b*^ All host strains used for phage isolation were *S. hyicus* strains (11).

^*c*^ The closest phage was identified based on the highest sequence similarity obtained from the BLASTn analysis.

^*d*^ The closest sequence for this phage is found in bacterial chromosome of *S. hyicus* strain NCTC10350 (GenBank: LS483304.1), 99.28% identity over 89% of the query cover.

**Fig 1:**
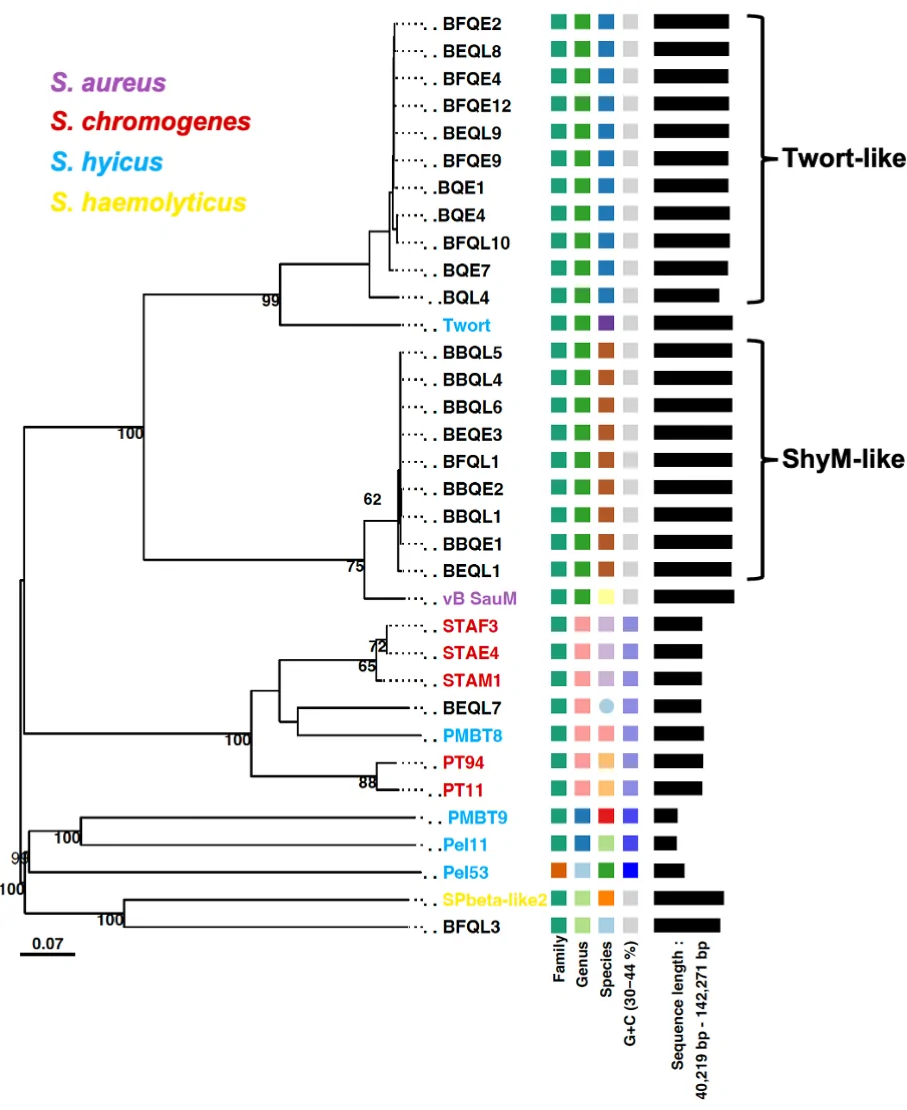
Phylogenetic tree of 22 new and 12 reference *Staphylococcus* spp. bacteriophages constructed using VICTOR based on whole-genome nucleotide sequences (GBDP method). Bootstrap values ≥ 60% are shown at the nodes. Colored boxes on the right indicate taxonomic assignments at the family, genus, and species levels inferred by VICTOR. Different colors are used to distinguish the corresponding taxonomic groups and automatically assigned by the software. Phage names shown in black represent newly isolated phages, whereas purple, red, blue, and yellow names indicate previously reported *S. aureus*, *S. chromogenes*, *S. hyicus*, and *S. haemolyticus* phages, respectively. Colored boxes on the right indicate taxonomic assignments at the family, genus, and species levels inferred by VICTOR. Phylogenetic analysis revealed that 20 phages clustered into two groups, Twort-like (11) and ShyM-like (9), whereas two phages (BEQL7 and BFQL3) were more closely related to other *Staphylococcus* spp. phages. All phages isolated in this study for which complete genome sequences were available were included in the phylogenetic analysis. Reference phage genomes were selected from the bacteriophages included in reference 11 and from the closest relatives identified through GenBank comparisons. GenBank accession numbers for the reference *Staphylococcus* spp. bacteriophages are as follows: Twort (MT151386), vB_SauM (MH107769), STAF3 (PV929973), STAE4 (PV929972), STAM1 (PV929971), PMBT8 (MK893987), PT94 (PP212903), PT11 (PP212900), PMBT9 (MW221967), Pel11 (PP971698), Pel53 (PP952070), and SPbeta-like2 (MT955612).

Genomic DNA was extracted using the DNeasy Blood and Tissue Kit (Qiagen, Toronto, ON, Canada) (12). Sequencing libraries were prepared and sequenced by Plasmidsaurus (Eugene, OR, USA) with Illumina NextSeq platform using paired-end 150-bp reads (11). Reads were quality-filtered using fastp (v0.23.4) (13), assembled with Shovill (v1.1.0) (14), and assessed for completeness using CheckV (v1.1.1) (15), with contigs receiving a completeness estimate of 100% considered complete genomes. Open reading frames were predicted using Pharokka (v1.7.5) (16) and functionally annotated with phold (v0.2.0) (17). Phylogenetic relationships were inferred using VICTOR (Fig. 1) based on the Mean-Genome-BLAST Distance Phylogeny (GBDP) method (18) using default parameters for all software mentioned above.

Genome sizes (Table 1) range from 83,716 to 139,223 bp, with guanine–cytosine (GC) contents between 30.22 and 31.72%. Genomes encode 160–248 predicted coding sequences, including two to seven lysis-related genes. Genome coverage ranges from 216.9 to 6,811.8×, and assemblies are classified as complete. Comparative genomic and phylogenetic analyses revealed similarity to previously described *Staphylococcus aureus*, *S. hyicus*, *S. chromogenes*, and *S. haemolyticus* phages (Fig. 1). Functional annotation using phold did not identify proteins associated with virulence, antimicrobial resistance, anti-CRISPR, or other known defense mechanisms.

Together, these genome data provide a valuable resource for understanding *Staphylococcus* phage diversity in a single farm and may support the development of phage-based approaches for the control of exudative epidermitis in swine.

## Data Availability

The GenBank accession numbers of the 22 bacteriophage genome sequences are listed in Table 1. The Sequence Read Archive data for the genomes are available under BioProject accession number PRJNA1465286.
